# The leading causes of death after burn injury in a single pediatric burn center

**DOI:** 10.1186/cc8170

**Published:** 2009-11-17

**Authors:** Felicia N Williams, David N Herndon, Hal K Hawkins, Jong O Lee, Robert A Cox, Gabriela A Kulp, Celeste C Finnerty, David L Chinkes, Marc G Jeschke

**Affiliations:** 1 Department of Surgery, University of Texas Medical Branch, 301 University Boulevard, Galveston, Texas 77555, USA; 2 The Shriners Hospitals for Children, 815 Market Street, Galveston, Texas 77550, USA

## Abstract

**Introduction:**

Severe thermal injury is characterized by profound morbidity and mortality. Advances in burn and critical care, including early excision and grafting, aggressive resuscitation and advances in antimicrobial therapy have made substantial contributions to decrease morbidity and mortality. Despite these advances, death still occurs. Our aim was to determine the predominant causes of death in burned pediatric patients in order to develop new treatment avenues and future trajectories associated with increased survival.

**Methods:**

Primary causes of death were reviewed from 144 pediatric autopsy reports. Percentages of patients that died from anoxic brain injuries, sepsis, or multi-organ failure were calculated by comparing to the total number of deaths. Data was stratified by time (from 1989 to 1999, and 1999 to 2009), and gender. Statistical analysis was done by chi-squared, Student's t-test and Kaplan-Meier for survival where applicable. Significance was accepted as *P* < 0.05.

**Results:**

Five-thousand two-hundred-sixty patients were admitted after burn injury from July 1989 to June 2009, and of those, 145 patients died after burn injury. Of these patients, 144 patients had an autopsy. The leading causes of death over 20 years were sepsis (47%), respiratory failure (29%), anoxic brain injury (16%), and shock (8%). From 1989 to 1999, sepsis accounted for 35% of deaths but increased to 54% from 1999 to 2009, with a significant increase in the proportion due to antibiotic resistant organisms (*P* < 0.05).

**Conclusions:**

Sepsis is the leading cause of death after burn injury. Multiple antibiotic resistant bacteria now account for the bulk of deaths due to sepsis. Further improvement in survival may require improved strategies to deal with this problem.

## Introduction

Burn injury is often followed by a profound hypermetabolic response that persists long after injury in those that survive [1,2]. The extent and duration of the response is related to the extent of the original burn injury sustained [2]. It is responsible for devastating muscle and protein catabolism, insulin resistance, and cardiac dysfunction that last for months after discharge, and significant growth retardation that impedes proper development [3,4]. Patients have supraphysiologic metabolic rates, multi-organ dysfunction, and increased inflammatory cytokines and acute phase proteins [4]. This response, which is mediated by 10- to 50-fold elevations in catecholamines, glucagon, and cortisol, leads to increases in morbidity and mortality [1,2,4,5]. Failure to attenuate the hypermetabolic response leads to irreparable damage and death.

The primary determinants of mortality from severe burn injury were described in 1997 as age, presence or absence of inhalation injury, and extent of burn [6]. However, recent advances in burn care including pharmacologic and non-pharmacologic modulations of the post-burn response, have led to significant improvements in morbidity and mortality [5]. Survival from a severe burn is no longer the exception, but the rule - even for those victims at the extremes of age [7,8]. Unfortunately, although patients with severe burns are more likely to survive, death still occurs. Thus, the aim of this study was to determine the predominant causes of death in severely burned pediatric patients in a single pediatric burn center, to guide physicians to focus on and evaluate new treatment avenues for clinical management to further improve survival.

## Materials and methods

### Medical records

The study was approved by the Institutional Review Board of the University of Texas Medical Branch and informed consent was obtained from patients, parents, or legal guardians prior to enrollment. Of more than 5200 children with burn injury admitted to the Shriners Hospitals for Children in Galveston, Texas, from July 1989 to July 2009, there were 145 in-hospital deaths. Autopsies were performed on all burned children that died at the hospital except for one patient (patient number 144) whose family refused due to religious reasons (99.3% of pediatric deaths underwent autopsy). All autopsies were performed at the Shriners Hospitals or at the adjacent University of Texas Medical Branch by two pathologists. In addition to the autopsy findings, pathologists reviewed the patients' hospital records and provided a summary of the clinical course. Patient demographics, characteristics, and clinical courses were recorded. The primary causes of death were assigned by the pathologist based on integration of the clinical information and the gross and microscopic findings from the autopsy. When present, herniation of the cerebellar tonsils across the tentorium cerebelli due to severe cerebral edema was considered to be the mechanism of brain death. In all cases in which brain death was declared based on clinical criteria but herniation was not identified at autopsy, neuropathologic examination demonstrated evidence of neuronal injury and necrosis sufficient to represent a cause of death. When no single immediate cause of death could be assigned, deaths were generally classified as being due to multiple-organ failure. These primary causes of death were obtained from the autopsy reports. The occurrence of inhalation injury and sepsis was also reviewed. Inhalation injury was diagnosed and confirmed with bronchoscopy. Charts were thoroughly reviewed for evidence of sepsis, including blood, tissue, and sputum culture results.

Percentages of patients that died from anoxic brain injuries, sepsis, pneumonia, or multi-organ failure due to circulatory shock were calculated by comparison with the total number of deaths. Data were stratified by time (from 1989 to 1999, and 1999 to 2009) to examine changes between time periods, and by gender to evaluate fundamental physiologic differences or treatment biases.

### Clinical care

Prior to death, all patients were admitted to the Shriners Hospitals for Children and all were treated in an identical manner by the same team of burn surgeons. Standard treatment included early excision of the burn wound, systemic antibiotic therapy, and continuous enteral feeding [6]. Standard treatment did not change significantly during the two decades studied. Within 48 hours of admission, each patient underwent total burn wound excision and grafting with autograft skin, allograft or both. Patients returned to the operating room when autograft donor sites healed and became available for reharvesting (usually 6 to 10 days). Sequential staged surgical procedures for repeat excision and grafting were undertaken until the wounds were healed. Each patient received enteral nutrition via naso-duodenal tubes with a high-protein (15 to 20%), high-carbohydrate (70 to 82%), low-fat (3 to 10%) feeding formula. Daily caloric intake was given at a rate calculated to deliver 1500 kcal/m^2 ^total body surface area (TBSA) burned plus 1500 kcal/m^2 ^TBSA. Enteral tube feeding was started at admission and continued at a constant rate until the wounds were healed. In general, patients remained at bed rest after excision and grafting procedures for four days. Thereafter, patients ambulated daily until the next excision and grafting procedure if clinically stable. Patient demographics (age, date of burn and admission, sex, burn size and depth of burn) and concomitant injuries, such as inhalation injury, sepsis, morbidity, and mortality were recorded. Inhalation injury was diagnosed by positive bronchoscopy associated with a positive history. Wound infection was defined by wound biopsies growing more than 10^5 ^colony forming units (CFU) per gram of tissue with the identification of the pathogen. Throughout acute hospitalization, we counted every incident of wound infection in which biopsies yielded more than 10^5 ^bacterial CFU/gram tissue, except subsequent recovery of the same bacterium in the same location, which was counted as one infection. Sepsis, multi-organ failure, and pneumonia were defined, as previously described [4,9-11]. Pneumonia was defined as the clinical diagnostic finding of a new and persistent infiltrate on chest x-ray, and a recent change in sputum or purulence in the sputum [9]. By definition, a diagnosis of sepsis and a change in sputum or new and persistent infiltrate on chest x-ray could be used for a clinical diagnosis of pneumonia [9].

Respiratory failure was defined as death caused by failure of the pulmonary system. It was categorized as death due to acute respiratory distress syndrome (ARDS), as defined clinically, diffuse alveolar damage (DAD) based solely on findings at autopsy, aspiration or asphyxia, or asthma attack. ARDS was clinically defined by meeting four criteria: acute onset; bilateral fluffy pulmonary infiltrates by x-ray; pulmonary artery wedge pressure less than 18 mmHg without evidence of left atrial hypertension; and a decrease in the ratio of partial pressure of arterial oxygen to fraction of inspired oxygen to 200 or less, indicating acute hypoxemia [12,13]. DAD by definition is the pathological diagnosis of ARDS [14]. It reflects injury to the pulmonary alveolar microvasculature and alveolar wall that leads to the exudation of fluid and plasma proteins that overwhelms the local lymphatic drainage [14]. Although diffuse alveolar damage was frequently recognized in cases of ARDS, there was an additional group of patients in whom histologic evidence of diffuse alveolar damage was considered sufficient to account for death even in patients who did not meet the clinical criteria for ARDS. Aspiration was defined as the inhalation of enteric contents or other material that compromised the airway.

Brain death was generally defined as the hypoxic or ischemic changes indicative of neuronal necrosis, as assessed by a neuropathologist, that were seen during the autopsy. The diagnosis of cerebral edema with herniation was generally made by clinical changes in the patients' neurological exam, confirmed radiographically, and confirmed again by the autopsy.

Statistical analysis was performed with chi-squared test, Student's t-test and Kaplan-Meier statistics where applicable. Significance was accepted as *P *< 0.05.

## Results

### Patient characteristics

**Figure 1:**
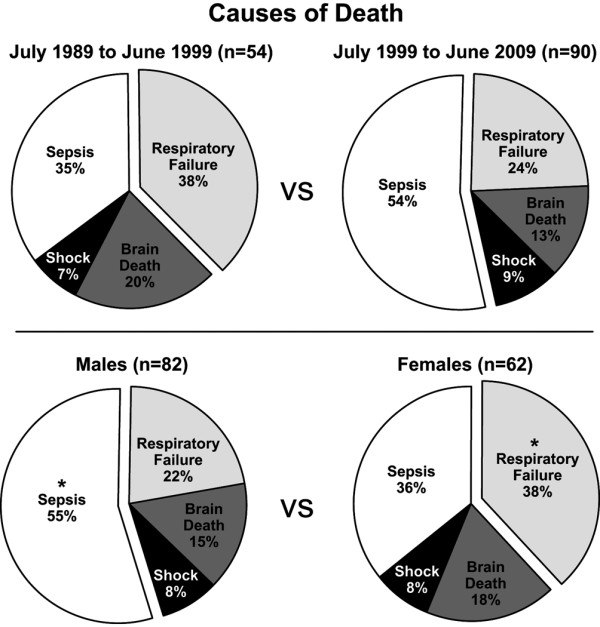
Cause of death stratified by decade and gender. More patients died of respiratory failure from July 1989 to June 1999, while more patients died from sepsis from July 1999 to June 2009. More male patients from all time points died of sepsis compared with females, while more female patients from all time points died of respiratory failure compared with males (*P < 0.05 compared with females).

Mortality for all acute burn admissions during this 20-year period was 145 of a total of 5260 patients, equal to 2.8% (Figure 1). The general patient characteristics are included in Table 1. Of patients who died, 71% had inhalation injury diagnosed clinically by bronchoscopy and autopsy findings (Table 1). From 1989 to 2009, the majority of pediatric burn patients was male, had suffered a flame burn injury and had 23% TBSA burn. Inhalation injury was present in 20% of all admitted burns.

**Table 1:** Patient characteristics

**Characteristic**	**Number of patients**
Total number of patients admitted from 1989 to 2009 (n)	5260
Total number of deaths (n)	145
Mortality (%)	2.8
Age of deceased (years)	7 ± 6
Total body surface area burned of deceased (%)	69 ± 23
Respiratory failure (%)	29
Brain death (%)	16
Shock (%)	8
Sepsis (%)	47*
Incidence of inhalation injury among deceased (%)	71
Time until death post-burn (days)	29 ± 50

Data presented as a percentage or average ± standard deviation.

* *P *< 0.05 compared with other causes of death.

Sepsis causes significantly more deaths than other etiologies.

### Respiratory failure

Respiratory failure accounts for 29% of all deaths. The average (standard deviation) TBSA was 61% ± 24%. ARDS assessed clinically accounts for 69% of the deaths caused by respiratory failure, which was significantly higher than all other causes of respiratory failure (14% due to aspiration or asphyxia, and 2% due to acute asthma attack; *P *< 0.05). DAD, the pathological correlate of ARDS, was present in many of these cases, but was considered to be the primary cause of death in an additional 14% of all respiratory deaths. Thus, 83% of respiratory deaths were due to ARDS. Nineteen percent of patients had TBSA less than 40%. Sixty-four percent of patients had inhalation injury. On average, patients lived 26 ± 35 days before death.

### Brain deaths

Brain injury accounted for 16% of all deaths. Anoxic brain injury accounted for 48% of the brain deaths after burn injury, while cerebral edema with herniation accounted for 52% of the brain deaths. The average TBSA was 62% ± 25%. Twenty-two percent of patients had TBSA less than 40%. Sixty-five percent of patients had inhalation injury. On average, patients lived 6 ± 5 days before death.

### Shock

Shock accounted for 8% of all deaths. The average TBSA was 67% ± 30%. Cardiac arrest secondary to hypovolemic circulatory shock accounts for 58% of deaths due to shock. In the remaining 42%, cardiovascular failure and shock were part of a picture of sterile multi-organ failure. Twenty-five percent of patients had TBSA less than 40%. Fifty percent of patients had inhalation injury. On average, patients lived 7 ± 10 days before death.

### Sepsis

**Figure 2:**
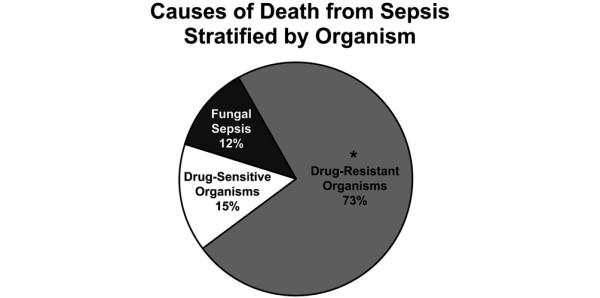
Cause of death from sepsis stratified by organisms. * *P *< 0.05 compared with other organisms. More septic patients died of drug-resistant organisms.

Sepsis accounted for 47% of all deaths. The average TBSA was 76% ± 18%. The organisms that caused sepsis are shown in Figure 2. Multi-drug resistant organisms, listed in Table 2, caused 73% of septic deaths (*P *< 0.05; Figure 2). Six percent of patients had TBSA less than 40%. Seventy-nine percent of patients had inhalation injury. On average, patients lived 43 ± 64 days before death.

**Table 2:** Sepsis stratified by decade and organisms

	July 1989 to June 1999	July 1999 to June 2009	*P *value
Deaths due to fungal sepsis (%)	26	6*	<0.05
Deaths due to sepsis from drug sensitive organisms (%)	32	8*	<0.05
Deaths due to sepsis from resistant organisms (%)	42	86*	<0.05

**Multi-Drug Resistant Organisms**			

*Klebsiella Pneumonia *(%)	25	2	NS
*Pseudomonas Aeruginosa *(%)	25	64	NS
*Acinetobacter baumannii *(%)	0	27	NS
MRSA (%)	12.5	5	NS
*Enterococcus faecalis *(%)	25	2	NS
*Enterobacter cloacae *(%)	12.5	0	NS

Data presented as average ± standard deviation.

* *P *< 0.05 compared with other causes of death.

MRSA = methicillin-resistant *Staphylococcus aureus*; NS = no significance.

There was a significant decrease in deaths due to fungal sepsis from July 1999 to June 2009 (*P *< 0.05; Table 2). There was a dramatic and statistically significant increase in deaths due to multi-drug resistant organisms from 1999 to 2009, compared with 1989 to 1999 (*P *< 0.05; Table 2). The percentage of deaths due to multi-drug resistant organisms increased from 42% to 86% (*P *< 0.05; Table 2). From 1999 to 2009, *Pseudomonas aeruginosa *was responsible for 64% of all deaths from multi-drug resistant organisms, followed by *Acinetobacter *species at 27% (Table 2).

### Multi-organ failure

Multi-organ failure was present in 51% of all deaths after burn injury.

### Changes over time

Mortality was similar between the decades (2.7% from July 1989 to June 1999, and 2.8% from July 1999 to April 2009). The average age of the non-survivors was 5 ± 5 years between July 1989 to June 1999, which was significantly younger than non-survivors from July 1999 to April 2009 (7 ± 6 years; *P *< 0.05). There were no significant differences in TBSA of non-survivors, incidence of inhalation injury, cause of death, or time until death. Respiratory failure was the primary cause of death from 1989 to 1999, while sepsis was the primary cause from 1999 to 2009, although this change was not statistically significant.

### Gender differences

There were no significant differences in age, percentage of deaths from brain injury, or shock, incidence of inhalation injury or time until death. Male non-survivors had significantly higher TBSA burns compared with female non-survivors (72% ± 23% versus 65% ± 23%, respectively; *P *< 0.05). Female non-survivors were more likely to die of respiratory failure (39% versus 22% in males; *P *< 0.05). Male non-survivors were more likely to die of sepsis (55% versus 36% in females; *P *< 0.05).

## Discussion

The mortality rate over this 20-year review was 2.8%. This is considerably lower than reported rates in the National Burn Repository (5.6%) [15]. More than 99% of all deaths that occurred at our institution had autopsies conducted. Only one patient who died at the institution (and is not included in this study) did not have an autopsy because of religious reasons. Low mortality rates, with high autopsy rates allowed us to investigate potential factors of clinical management that are correctable and could lead to improved survival.

Acute lung injury or ARDS accounted for 40% to 50% of all deaths among the critically ill [9-11,16] ARDS is a clinical diagnosis. Sixty-nine percent of patients that died from respiratory failure, died because of ARDS. Although the methods used for management of patients with ARDS has changed dramatically between 1989 and 1999 and 1999 and 2009, the mortality rate remained the same, whether or not there was clinical evidence of smoke inhalation injury. In addition, the breakdown of respiratory failure demonstrates the potential overlap of clinical diagnoses. Many patients that died of ARDS had evidence of pneumonia, and also demonstrated pathological evidence of DAD. The one patient that died of an acute asthma attack also had ARDS, but it was the asthma attack that was the fatal event. Respiratory compromise can be a global problem in burn patients as mucus accumulates in distal parenchyma and bronchioles influenced by an increased secretory state of submucosal glands and decreased mucociliary function secondary to resuscitation efforts, and mechanical ventilation [17]. This physiologic change underscores the fact that overlapping diagnoses may contribute to death. Cases in which there were overlapping diagnoses, patients were placed in the category corresponding to the primary cause of death at autopsy. Thus, a patient may have pneumonia, but the primary cause of death was an anoxic brain injury. On the otherhand, patients may have confirmation of anoxic brain injury at autopsy, but the primary cause of death was overwhelming sepsis.

Patients diagnosed with ARDS were treated in concordance with the guidelines outlined in the ARDSNET trial in order to improve mortality [12]. Although this trial did not include pediatric burn patients, we had better outcomes with lower tidal volumes and lower plateau pressures [12]. The data suggest that the decrease in respiratory deaths from 1999 to 2009 may be associated with these more gentle, supportive ventilator practices.

Only 14% of deaths with burns were in patients with burns less than 40% of their TBSA (minor burns). Of note, 22% of patients that died from brain injury had minor burns. In addition, a quarter of patients that died from shock had burns encompassing less than 40% TBSA. These etiologies are attributed to delays in care or resuscitation, or deficient fluid resuscitation. Regardless of the extent of injury, airways for these particular patients were not obtained or maintained to ensure survival. A prior study looking at the determinants of mortality in severely burned patients underscored the relation between delays in resuscitation and increased mortality [6]. This study shows that this holds true despite the size of burn.

**Figure 3:**
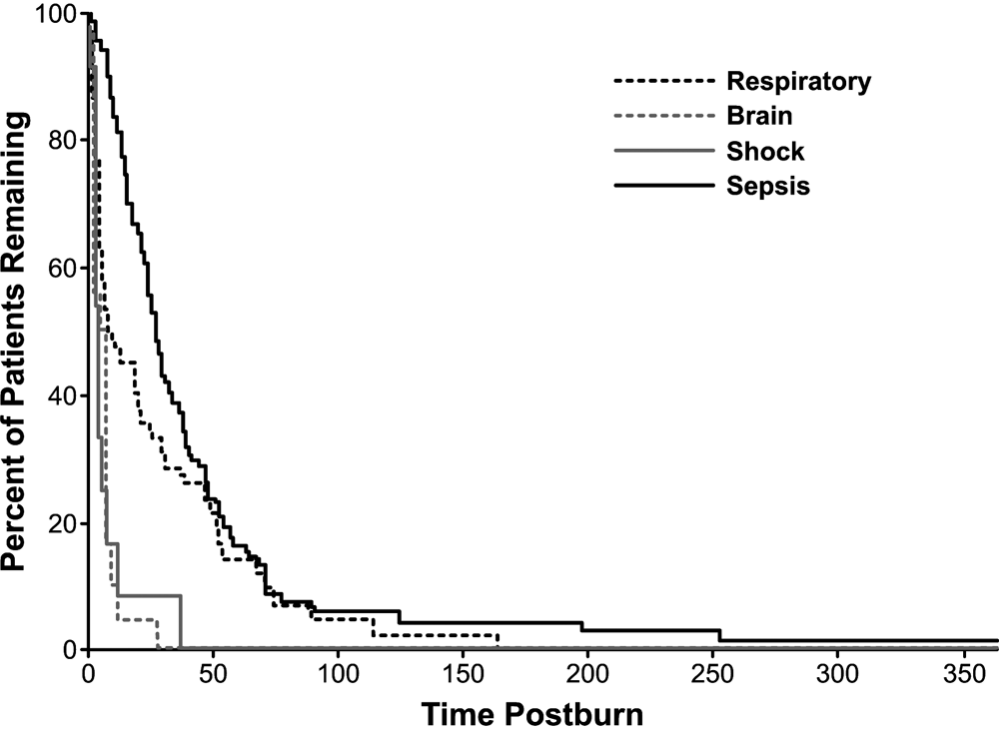
Percent of patients remaining for the different causes of death. * *P *< 0.05. Patients that died of sepsis lived longer until death compared with patients that died of shock, brain death, or respiratory failure.

Patients who died of sepsis had longer times until death compared with patients who died of other etiologies (Figure 3). This was significantly longer than in the anoxic brain injury, shock, and respiratory failure groups (*P *< 0.05). Patients with respiratory failure had the second longest time until death, but this was not statistically significant. Life may have been prolonged in this patient population with the use of mechanical ventilation. Further investigation needs to be conducted to see if ARDS in this patient population was due, at least in part, to ventilator-associated pneumonia.

Multi-organ failure was present in over half of all deaths after burn injury. It is caused here by sepsis, hypoxia, hypovolemia, and shock. Again, these etiologies can be attributed to delays and deficiencies in care and resuscitation.

The most notable finding in this review was the effect of multi-drug resistant organisms on long-term survival. From 1989 to 1999, only 42% of patients died from sepsis from multi-drug resistant organisms and 25% of patients had *Pseudomonas *as the organism responsible. From 1999 to 2009, 86% of patients that died from sepsis, died from multi-drug resistant organisms and 64% of those patients had *Pseudomonas *as the organism responsible. Sepsis deaths from *Acinetobacter *did not arise in our institution until 1999 to 2009, and that organism was associated with the demise of 27% of patients with multi-drug resistant deaths. Although this was a substantial increase, it was not a statistically significant increase due to the sample size. Despite advances in anti-microbial therapies, the number of deaths associated with multiple antibiotic resistant organisms has increased. The incidence of invasive fungal infections decreased in the second decade. This finding is significant as invasive fungal infections lead to increased morbidity and mortality [18]. The decline in incidence of death due to fungal infection can be attributed to development of more effective antifungal therapies during the time period studied. In general, strategies to prevent infection, such as early excision and grafting, aggressive anti-microbial therapy, including the use of colistin, and early enteral feedings improve survival [14,15,17-21]. On the other hand, widespread use of aggressive anti-microbial therapies has led to increased colonization of pathogens that have resistance to current therapies [20,22]. In addition, faulty contact isolation practices propagate spreading the organism from one patient to the next [19,20]. With any signs of infection, patients were cultured, including blood, sputum, urine and tissue, and started on broad-spectrum antibiotics (covering for Gram-negative and Gram-positive organisms, fungi and parasites). Once cultures and sensitivities had been identified, therapy was tailored to these organisms. Despite these practices drug-resistant organisms remain a threat and challenge in the burn unit. The development and strengthening of pathogens to resist anti-microbial therapy are linked to the dramatic increase in the percentage of sepsis-related deaths in our institution.

A recent study showed that female patients had a more attenuated hypermetabolic and inflammatory response compared with males [22]. Another issue raised by these findings relates to the aggressiveness with which we treat male and female patients. Female patients were more likely to die of respiratory failure, than any other cause, but had a lower incidence of inhalation injury. In addition, female patients had a lower incidence of sepsis. The question remains of whether female patients were more aggressively resuscitated, leading to fluid overload and need for mechanical ventilation, or if they received more aggressive anti-microbial therapy.

In this study, all but one of all patients who died had an autopsy performed, thus, we suggest that these findings are representative of clinical care and management, despite the fact that autopsies are known to disagree with clinical diagnoses in up to 40% of cases [23,24]. Patients, regardless of burn size, age, or point of origin have become more likely to survive a burn injury during the past 20 years. Those that did not survive had some evidence of delays or deficits in resuscitation with either airway management or volume leading to burn shock. The progression to multi-organ failure from shock was prolonged due to the extensive physiologic reserve and cardiac resilience that are characteristic of children. The development of sepsis significantly contributed to the demise of patients with and without the emergence of multi-drug resistant organisms.

The main focus of this study was on the single primary immediate cause of death. Burn trauma is a complicated injury that causes profound physical and physiologic derangements. The clinical course for these patients is also complicated. For example, many patients died with anoxic brain injury but that injury was not the primary cause of death. Furthermore, many patients died with burn wound infections due to multi-resistant organisms, but these infections were not the primary cause of death. Some patients died with derangements in multiple organ systems, which led to their demise.

## Conclusions

On some level, most burn deaths may be preventable with better airway management and more aggressive but precise resuscitative efforts. However, sepsis, due to multi-drug resistant organisms, may continue to impede efforts to increase survival if we cannot develop strategies to fight these organisms that go beyond the surgical and clinical techniques that have been implemented already. The data suggested that while most severe burn injuries are survivable, delays in resuscitation, inadequate resuscitation (leading to inadequate tissue perfusion), poor airway management, and inappropriate or inadequate anti-microbial coverage lead to increased morbidity and mortality in our patients. Advances and improvements in airway management, and resuscitative efforts have led to a decrease in deaths caused by those deficiencies, but deaths due to multi-drug resistant organisms still represent a challenge. Also, more studies need to be conducted to examine the potential gender differences in the response to sepsis, and the response to therapy. Further studies will investigate the proteomic and genomic changes post-burn in all patients in order to identify patients at increased risk of becoming recalcitrant to treatment modalities for sepsis, multi-organ failure, and persistent respiratory failure.

## Key messages

• Respiratory failure and sepsis are the leading causes of death in severely burned pediatric patients.

• Deficiencies or delays in resuscitation increase risk of death after burn despite the size of burn injury.

• Multi-organ failure is present in over 50% of all deaths after burn injury.

• Future studies are needed to outline genomic or proteomic pathways that predispose patients to different outcomes.

## Abbreviations

ARDS: acute respiratory distress syndrome; cfu: colony forming units; DAD: diffuse alveolar damage; TBSA: total body surface area.

## Competing interests

The authors declare that they have no competing interests.

## Authors' contributions

FNW performed the retrospective review, wrote the manuscript, and performed the statistical analysis. DNH outlined the design of study, drafting the manuscript, and was clinically responsible for the patients. HKH participated in the design of the study, drafting the manuscript, and performed the autopsies. JOL participated in the design of the study, drafting the manuscript, and was clinically responsible for the patients. REC participated in the design of the study, and drafting the manuscript. GAK participated in the design of study and retrospective review. CCF participated in the design of the study, and drafting of the manuscript. DLC participated in the design of the study, drafting the manuscript, and performed the statistical analysis. MGJ participated in the design of the study, drafting the manuscript, and was clinically responsible for the patients. All authors read and approved the final manuscript.
